# ‘Fatigue makes cowards of us all’

**DOI:** 10.1113/EP091111

**Published:** 2023-02-06

**Authors:** David C. Poole, Shunsaku Koga

**Affiliations:** ^1^ Department of Kinesiology Kansas State University Manhattan Kansas USA; ^2^ Department Anatomy & Physiology Kansas State University Manhattan Kansas USA; ^3^ Applied Physiology Laboratory Kobe Design University Nishi‐ku Kobe Japan

**Keywords:** exercise performance, exhaustion, muscle oxygenation, oxygen uptake kinetics, quadriceps EMG, severe‐intensity exercise, slow component

1

*Fatigue makes cowards of us all*.

General George S. Patton Jr, 1944

Whether it is the physical performance of soldiers on the battlefield, as for Lt. General George S. Patton Jr (1947), athletes on the American football field, as for legendary Green Bay Packers’ coach Vince Lombardi, patients whose quality of life is eroded by physical incapacity or the rest of us, fatigue impacts us all. Understanding the mechanistic bases for exercise intolerance, in health and disease, is thus a key objective for integrative physiologists and clinician scientists. However, this process was compromised throughout much of the 20th century by failure to recognize and appreciate the additional energy cost invoked by exercise above the lactate threshold. Thus, for heavy and severe‐intensity exercise a secondary, slowly developing (hence ‘slow component’) of the oxygen uptake (${\dot{V}}_{O_{2}}$) kinetics, which constitutes an extra energy cost, is superimposed upon the fundamental fast ${\dot{V}}_{O_{2}}$ kinetics (reviewed by Gaesser & Poole, 1996). In the extreme, this ${\dot{V}}_{O_{2}}$ slow component is of extraordinary magnitude (1–1.5 L O_2_/min) and, in the severe domain, drives ${\dot{V}}_{O_{2}}$ to its maximum (i.e., ${\dot{V}}_{O_{2}\max}$) and augers imminent exhaustion. Experimental manipulation of the ${\dot{V}}_{O_{2}}$ slow component by altered work output or O_2_ transport markedly impacts exercise tolerance, demonstrating that the two are inextricably intertwined, and it is therefore appropriate that this relationship provided the foundation for the recent paper by do Nascimento Salvador et al. (2023) investigating the mechanisms of fatigue.

Fatigue and/or exhaustion present complex problems that have a context and task dependence and might, in part, require future technological advances to address fully. Notwithstanding these challenges, the paper by do Nascimento Salvador et al. (2023) provides some pertinent, new and intriguing insights. Specifically, using non‐exhaustive cycle ergometry bouts in the moderate, heavy and very heavy/severe exercise intensity domains, their data support an association between vastus lateralis muscle oxidative capacity, tissue O_2_ saturation index, recruitment of additional type I and II motor units (rectus femoris) and muscle fatigue. As noted previously by others (e.g., Cannon et al., 2011), do Nascimento Salvador et al. (2023) found a temporal dissociation between muscle fatigue (early) and the ${\dot{V}}_{O_{2}}$ slow component (later), concluding that fatigue is a prerequisite for the ${\dot{V}}_{O_{2}}$ slow component. This conclusion embodies the principle that ‘${\dot{V}}_{O_{2}}$ kinetics sow the seeds of exercise intolerance’, acknowledging that exercise performance is influenced not only by the fundamental fast ${\dot{V}}_{O_{2}}$ kinetics but also by the ${\dot{V}}_{O_{2}}$ slow component, which itself might originate close to exercise onset (i.e., within 2–3 min of exercise onset).

Unravelling the mechanisms of fatigue and exhaustion during large muscle mass exercise, such as cycling, presents the investigators with significant challenges that are compounded by the following factors. First, there are simultaneous changes in many of the primary variables of interest (e.g., muscle high‐energy phosphates, muscle acid–base balance, glycogen, muscle fibre recruitment, neuromuscular transmission and central activation), in addition to blood acid–base and hormone/signalling molecules, ${\dot{V}}_{O_{2}}$, ventilation, body temperature and perceived exertion. Previously, correlations were established between the temporal profiles of the ${\dot{V}}_{O_{2}}$ slow component and corollary processes such as ventilation, catecholamines and, especially, blood lactate concentration (Gaesser & Poole, 1996). However, in keeping with the good science principle that correlations do not prove causation, prospective studies in animals and humans resolved that the vast majority of the ${\dot{V}}_{O_{2}}$ slow component occurred within the exercising limb muscles (not the respiratory muscles) and thus was not dependent upon blood lactate or noradrenaline concentrations; at least acting at remote sites (reviewed by Gaesser & Poole, 1996). Second, do Nascimento Salvador et al. (2023) acknowledge substantial heterogeneity between the two muscles (vastus lateralis and rectus femoris) that they investigated. This is certainly true with respect to spatial and temporal activation (EMG) and muscle oxygenation profiles. Indeed, Okushima et al. (2015) have, during heavy‐intensity cycling exercise, resolved not only oxygenation (O_2_ delivery–${\dot{V}}_{O_{2}}$ matching) heterogeneity among quadriceps muscles but also spatially across individual muscles and deep versus superficial muscle.

In conclusion, the temporal (but not quantitative) dissociation of fatigue from the ${\dot{V}}_{O_{2}}$ slow component by do Nascimento Salvador et al. (2023) reinforces previous observations valuably. Moreover, their new associations of muscle oxidative capacity, tissue O_2_ saturation index, recruitment of additional type I and II motor units (rectus femoris) with muscle fatigue, although only correlative, are exciting and will hopefully provide imperative and direction for prospective mechanistic investigations.

## AUTHOR CONTRIBUTIONS

Both authors approved the final version of the manuscript and agree to be accountable for all aspects of the work in ensuring that questions related to the accuracy or integrity of any part of the work are appropriately investigated and resolved. All persons designated as authors qualify for authorship, and all those who qualify for authorship are listed.

## CONFLICT OF INTEREST

None declared.

## FUNDING INFORMATION

No funding was received for this work.
